# Dietary phytochemical PEITC restricts tumor development via modulation of epigenetic writers and erasers

**DOI:** 10.1038/srep40569

**Published:** 2017-01-12

**Authors:** Jung Eun Park, Yang Sun, Sai Kiang Lim, James P. Tam, Matthijs Dekker, Hong Chen, Siu Kwan Sze

**Affiliations:** 1 School of Biological Sciences, Nanyang Technological University, 60 Nanyang Drive, 637551 Singapore; 2 Institute of Medical Biology, A*STAR, 8A Biomedical Grove, #05-05 Immunos, 138648 Singapore; 3 Food Quality and Design Group, Department of Agrotechnology and Food Sciences, Wageningen University, PO Box 8129, 6700 EV Wageningen, Netherlands; 4 Department of Food Science and Human Nutrition, University of Illinois, 472 Bevier Hall, 905 S. Goodwin, Urbana IL 61801, USA

## Abstract

Dietary intake of bioactive phytochemicals including the cruciferous vegetable derivative phenethyl isothiocyanate (PEITC) can reduce risk of human cancers, but possible epigenetic mechanisms of these effects are yet unknown. We therefore sought to identify the molecular basis of PEITC-mediated epigenetic tumor restriction. Colon cancer cells treated with low-dose PEITC for >1 month exhibited stable alterations in expression profile of epigenetic writers/erasers and chromatin-binding of histone deacetylases (HDACs) and Polycomb-group (PcG) proteins. Sustained PEITC exposure not only blocked HDAC binding to euchromatin but was also associated with hypomethylation of PcG target genes that are typically hypermethylated in cancer. Furthermore, PEITC treatment induced expression of pro-apoptotic genes in tumor cells, which was partially reversed by overexpression of PcG member BMI-1, suggesting opposing roles for PEITC and PcG proteins in control of tumor progression. These data demonstrate that PEITC regulates chromatin binding of key epigenetic writers/erasers and PcG complexes to restrict tumor development.

Human cancers are highly diverse, exhibit complex aetiology, and remain extremely challenging to treat in the clinic. As the total burden of disease and associated healthcare costs continue to rise, attention is now increasingly turning to methods of cancer prevention rather than cure^1^,^2^. Various epidemiological studies have suggested that a promising approach could be to increase consumption of naturally-occurring bioactive dietary compounds (BDCs) which have been reported to modulate the epigenome and exert potent effects on cellular gene expression^3^,^4^. While there is strong evidence that cancer risk is reduced by dietary intake of BDCs derived from vegetables such as those in the brassica family^5^,^6^, an epigenetic basis of these effects remains elusive.

Rapid technological progress in recent decades has finally enabled investigators to begin to define the epigenetic characteristics of different cancers and identify the environmental factors that promote disease^7^. For example, we now appreciate that aberrant patterns of DNA methylation and/or histone modification contribute to the induction of chromosomal instability^8^ and inactivation of key tumor suppressor genes including *CDKN2A, MLH1, CDH1*, and *VHL*^9^,^10^. Accordingly, the role of key epigenetic modifiers in the control of tumorigenesis has come under increasing scrutiny, with particular focus on identifying the pro- or anti-cancer epigenetic marks induced by DNA methyltransferases (DNMTs), histone acetyltransferases (HATs), and histone deacetylases (HDACs)^11^,^12^,^13^. Given that chromatin conformation and epigenetic profile are highly plastic, it has been proposed that therapeutic targeting of epigenetic regulators could represent an effective strategy for cancer treatment^14^.

Current epigenetic therapies for cancer include inhibitors of the enzymes HDACs and DNMTs that aim to restore a ‘healthy’ epigenetic state via the removal/reversal of cancer-associated epigenetic marks^13^,^15^,^16^. However, HDACs and DNMTs are also essential for the downstream maintenance of chromatin structure in the steady-state, hence non-selective inhibition of these enzymes can induce serious adverse effects that disrupt normal cell function^17^,^18^. Recently, dietary phytochemicals such as indicaxanthin (Ind), genistein, and several flavones such as apigenin, chrysin, and luteolin, have been reported to decrease DNA methylation level by inhibiting DNMT activities^19^,^20^,^21^. Interestingly, these flavones and Ind showed a direct interaction with DNMTs, which assessed by molecular modeling, showed possibility as good alternatives instead of chemically synthesized DNMT inhibitors^20^,^21^. Therefore, a more promising approach could be to identify how dietary intake of bioactive phytochemicals can promote and maintain ‘anti-tumor’ epigenetic profiles in the consumer, potentially leading to low-cost dietary interventions that protect a large fraction of the global population against cancers. Indeed, dietary factors are already known to exert a major environmental influence on the epigenetic profile of human cells^3^, and several BDCs are associated with chemoprevention when consumed in sufficient quantities, including the green tea derivative epigallocatechin-3-gallate (EGCG), the soy bean derivative genistein, and cruciferous vegetable derivative sulforaphane (SFN) and phenethyl isothiocyanate (PEITC)^5^. The most potent of these compounds is PEITC, which can modulate expression of many cancer-related genes including those involved in antioxidant responses, resistance to apoptosis, cell cycle regulation, or metastasis^22^,^23^. PEITC is formed upon hydrolysis of the parent glucosinolate gluconasturtiin, which occurs in many *Brassica rapa* varieties that are part of the human diet like Chinese cabbage, Pak Choi and turnips^24^. PEITC-mediated anticancer effects were demonstrated by induction of apoptosis or cell cycle arrest in PEITC treated cancer cells such as HER2-positive breast cancer cells and pancreatic cancer cells^25^,^26^. While the anti-tumor effects of these BDCs are thought to be partially mediated at the level of the epigenome, there is currently only limited understanding of the molecular mechanisms that integrate environmental signals and re-shape the epigenome in human cells. Future development of effective dietary regimens for cancer prevention will therefore require a better understanding of BDC effects on DNA methylation, histone modification, and/or chromatin remodeling in developing tumors.

In the current study, we investigated how PEITC exposure modifies epigenetic profiles in a tumor cell line model of colorectal cancer (CRC), which is the third most common type of cancer and the second leading cause of cancer-related death worldwide^27^. CRC is known to arise from an accumulation of genetic and epigenetic defects, but while the primary gene mutations predisposing to CRC have been well-documented, the epigenetic mechanisms that promote disease are not fully understood^28^,^29^. We therefore conducted global quantitative profiling of chromatin-associated proteins in PEITC-treated CRC cells together with microarray assessment of DNA methylation patterns to determine how this dietary phytochemical restricts tumor development at the epigenetic level. Collectively, our data reveal that PEITC inhibits cancer progression by modifying expression patterns of epigenetic writers/erasers, promoting hypomethylation of PcG target genes, and blocking HDAC binding to euchromatin in tumor cells. By improving our understanding of how BDCs modify the chromatin-associated proteome, we can begin to exploit these effects to prevent or even reverse oncogenic changes in the epigenome to more effectively treat human cancers.

## Results

### Long-term PEITC exposure modifies cancer cell phenotype and epigenetic profile

**Figure 1:**
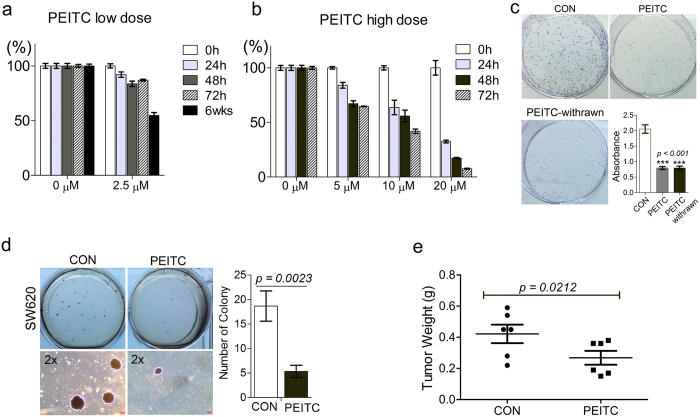
Prolonged exposure to low-dose PEITC reduces growth and tumorigenic potential of colon cancer cells both *in vitro* and *in vivo*. (**a,b**) MTT assay determination of SW620 cell viability after treatment with low-dose PEITC (**a**) or high-dose PEITC (**b**) for the indicated times. Error bars represent mean ± s.d. (**c**) Colony formation by SW620 cells after 6 weeks treatment with 2.5 μM PEITC (SW620-PEITC) followed by subsequent culture in the presence (PEITC) or absence (PEITC-withrawn) of PEITC treatment for 21 days. SW620 cells exposed to DMSO vehicle only with the corresponding treatment duration served as the control (SW620-CON). Error bars represent mean ± s.d. (**d**) Anchorage-independent growth of SW620 cells after 6 weeks treatment with 2.5 μM PEITC (PEITC) or DMSO-only vehicle control (CON). The number of colonies formed in soft agar was quantified after 21 days incubation. Error bars represent mean ± s.d. (**e**) Delayed growth of SW620-PEITC-derived tumors in a mouse xenograft model. SW620 cells were treated with either DMSO only (CON) or 2.5 μM PEITC (PEITC) for 6 weeks prior to s.c. injection into the left or right flank of recipient nude mice. Tumor burden was measured by assessment of gross weight in independent duplicate experiments. Statistical differences between groups were determined using Student’s t-test (mean ± s.d., n = 12, *p = *0.0212*).

Prolonged exposure to BDCs confers stable and transferrable epigenetic changes that may improve host protection against tumors, so we studied the effects of sustained PEITC exposure on the oncogenic properties of colorectal cancer cells. To do this, we cultured SW620 colon cancer cells for a total 6 weeks duration either in the presence 2.5 μM PEITC (SW620-PEITC) or 0.01% DMSO vehicle-only (SW620-CON). As shown in Fig. 1a, SW620 cells subjected to long-term PEITC exposure exhibited a 40% decrease in viability compared with cells exposed to DMSO vehicle only. These effects were not seen when SW620 cells were treated with the same dose of PEITC for ~72 h duration (86% viability relative to control cells), indicating that 2.5 μM PEITC is not acutely cytotoxic in short-term cultures (Fig. 1a,b and Supplementary Fig. 1). We next investigated the effects of prolonged exposure to low-dose PEITC on SW620 cell growth rate in clonogenic assays (Fig. 1c) and assessed tumorigenic potential both in soft agar colony formation assays *in vitro* (Fig. 1d) and in a mouse xenograft model *in vivo* (Fig. 1e). SW620-PEITC cells exhibited impaired colony formation and decreased anchorage-independent growth compared with control cells even when assessed 21 days after PEITC withdrawal, indicating that this treatment induces durable phenotypic changes that are likely heritable by successive generations of tumor cells. Accordingly, when we performed subcutaneous (s.c.) injection of SW620-CON or SW620-PEITC cancer cells into either the left or right flank of NCr nude mice, we observed a significant delay in growth of tumors derived from the PEITC-treated cells compared with controls (*p* = *0.0212*; Fig. 1e and Supplementary Fig. 2). The mean weight of tumors generated by SW620-PEITC cells was 63.6% of that generated by SW620-CON cells assessed at the same time point, indicating that long-term exposure to low concentration of PEITC can potently restrict tumor growth *in vivo*.

**Figure 2:**
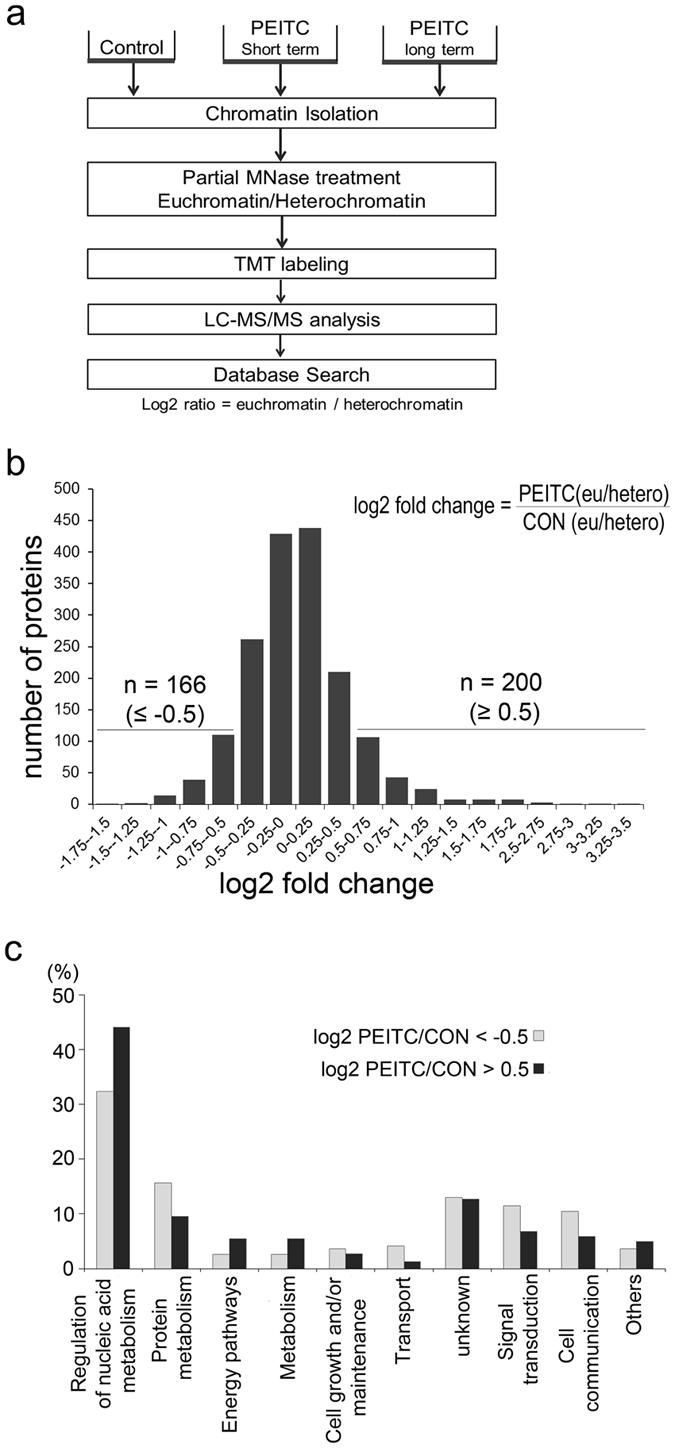
Tandem Mass Tag analysis of chromatome composition and gene ontology. (**a**) Schematic outline of LC-MS/MS analytical method. Chromatin-binding proteins were isolated by partial MNase treatment and subsequently labeled with TMT tags. (**b**) Histogram representation of changes in protein binding to either euchromatin or heterochromatin in SW620 cells after long-term PEITC treatment (relative to vehicle-only control). Differentially regulated proteins displaying log2 fold-change ≤ −0.5/ ≥ 0.5 are indicated. (**c**) GO enrichment analysis of the corresponding biological processes is shown in the bar graph.

We next sought to determine whether the ability of PEITC conditioning to restrict tumor growth was achieved via epigenetic modifications that influence gene expression. We therefore used a quantitative proteomic approach to assess whether PEITC treatment of SW620 cells alters the composition of the ‘chromatome’ i.e. the selection of proteins that are bound to regions of euchromatin or heterochromatin. To do this, we purified chromatin from either SW620-CON or SW620-PEITC cells and subjected this to partial digestion by micrococcal nuclease (MNase) which can access the relatively open structure of euchromatin but not the closed conformation of heterochromatin thereby sequentially extracting euchromatin- and heterochromatin-associated proteins^30^. Next, we subjected the protein samples to trypsin digestion and then extracted and labelled the constituent peptides with 6-plex isobaric Tandem Mass Tags (TMT) for LC-MS/MS analysis^31^ (Fig. 2a). Protein identification and quantitation were performed using Mascot database search software via the Proteome Discoverer™ 1.4 interface (specific search criteria are described in the methods section and supplementary data). A total of 2604 proteins were identified based on at least two unique tryptic peptides and false discovery rate (FDR) < 1%, of which 1889 proteins (72.5%) were reported to originate in the nucleus. Protein shifts from euchromatin to heterochromatin association were calculated as the ratio of abundance in PEITC-treated cells relative to control cells (with log2 fold change ≤ −0.5 and ≥0.5 assigned as cut-off values; Fig. 2b). This approach revealed that long-term PEITC treatment exerts a selective influence on chromatome composition, with the majority of proteins (80.7%) displaying negligible log2 fold change in treated cells (between −0.5 and 0.5; Fig. 2b). However, a total of 366 proteins (19.3% of the total nuclear proteins) were observed to have altered their chromatin binding topology relative to the control sample (Supplementary Table 1). Among these, 200 proteins were enriched in euchromatin after PEITC-treatment (log_2_PEITC/CON ≥ 0.5) whereas 166 proteins had been redistributed to regions of heterochromatin (log_2_PEITC/CON ≤ −0.5). Next, the PEITC-modulated proteins (n = 366) were subjected to gene ontology (GO) analysis and functional classification as shown in Fig. 2c.

### PEITC modulates histone epigenetic regulators in colon cancer cells

**Figure 3:**
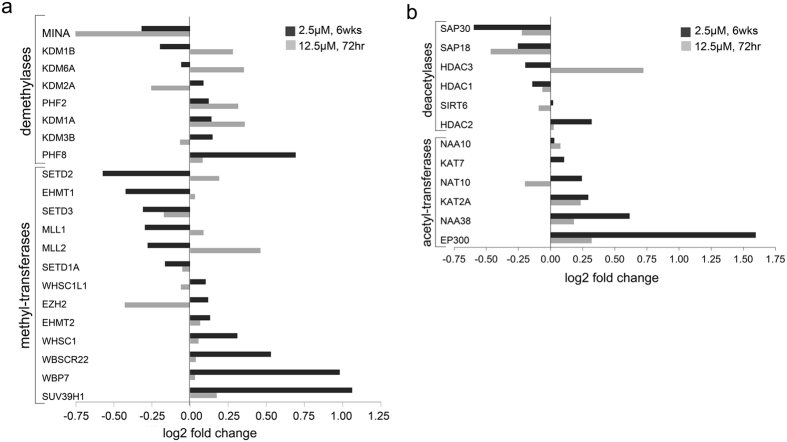
PEITC modulates histone epigenetic regulators in colon cancer cells. (**a,b**) Modulation of histone methyl transferases and demethylases (**a**) or acetyltransferases and deacetylases (**b**) in SW620 cells after short- or long-term exposure to low-dose PEITC. Differential protein binding to either euchromatin or heterochromatin in SW620 cells after 6 weeks or 72 hr PEITC treatment was calculated by log_2_(PEITC-6wks/CON) or log_2_(PEITC-72 hr/CON).

In order to identify key mediators of chromatome modulation by PEITC, we next focused our attention on the distribution of major epigenetic regulators identified in the LC-MS/MS data. We observed that PEITC treatment modulated the chromatin association patterns of several major groups of epigenetic writers and erasers^32^, including polycomb repressive complexes (PRCs), histone lysine methyltransferases (HMTs), histone acetyltransferases (HATs), histone deacetylases (HDACs), and lysine demethylases (Fig. 3). Among these were several HATs that were redistributed to regions of euchromatin in PEITC-treated cells, such as EP300 (E1A binding protein p300), NAA38 (N-Alpha-Acetyltransferase 38), KAT2A (lysine acetyltransferase 2A), and NAT10 (N-acetyltransferase 10). Conversely, multiple HDACs displayed preferential binding to heterochromatin upon PEITC exposure, including HDAC1, HDAC3, SAP18 (sin3 associated protein 18), and SAP30 (sin3 associated protein 30), suggesting that PEITC may block HDAC binding to open regions of active euchromatin. Strikingly, short-term PEITC treatment was instead associated with only mild effects on the distribution of histone epigenetic regulators in SW620 cells, suggesting that long-term exposure is required to induce stable and heritable changes in epigenetic profile of tumor cells.

**Figure 4:**
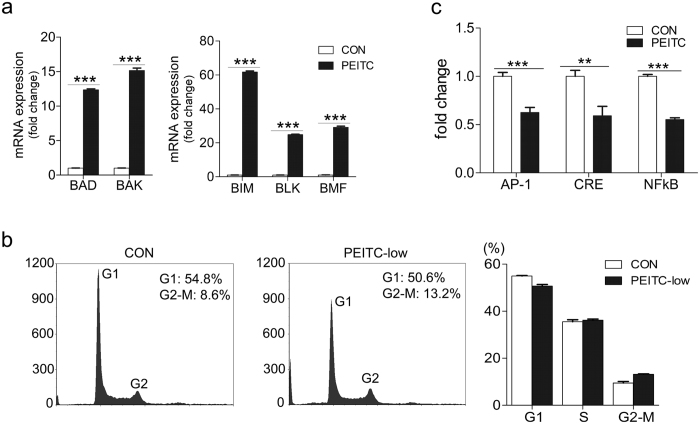
PEITC treatment is associated with tumor upregulation of pro-apoptotic genes. (**a**) Up-regulation of pro-apoptotic gene expression in SW620-PEITC cells compared with SW620-CON cells as determined by qRT-PCR. Error bars represent the mean ± s.d., n = 3. (**b**) Flow-cytometry analysis of propidium iodide-stained SW620 cells treated with 2.5 μM PEITC for 6 weeks (or DMSO-only vehicle control). Shown are representative data from one of three independent experiments conducted. (**c**) SEAP reporter assay quantification of AP-1, CRE, and NFkB signaling pathway activity in SW620 cells treated with 2.5 μM PEITC or DMSO only (CON) for 6 weeks duration. Error bars represent the mean ± s.d., n = 3. Statistical differences between groups were determined using Student’s t‐test (**p < *0.01*; ***p < *0.001*).

We next investigated the phenotypic and functional consequences of these PEITC-induced chromatome changes in terms of tumor cell apoptosis and cell cycle arrest using quantitative real-time PCR (qRT-PCR) and flow-cytometry. As shown in Fig. 4a, PEITC treatment of SW620 cells was associated with transcriptional induction of the *BAD, BIM, BLK*, and *BMF* genes which encode pro-apoptotic BH3 domain proteins, as well as the multi-domain pro-apoptotic gene *BAK*^33^, whereas cell cycle distribution was comparable with that of SW620-CON cells (Fig. 4b). We also assessed PEITC effects on the activity of key tumorigenic signaling pathways in colon cancer cells using a secreted alkaline phosphatase (SEAP)-based reporter assay^34^. Unlike SW620-CON cells, tumor cells treated with PEITC displayed impaired signaling via AP-1 (activator protein 1), CRE/CREB (cAMP response elements), and NFkB pathways (Fig. 4c). Together, these data indicate that long-term exposure to low-dose PEITC is associated with durable changes in chromatome distribution, activation of pro-apoptotic genes, and deficits in tumorigenic signaling pathways.

### PEITC induces distinct patterns of DNA methylation in colorectal cancer cells

**Figure 5:**
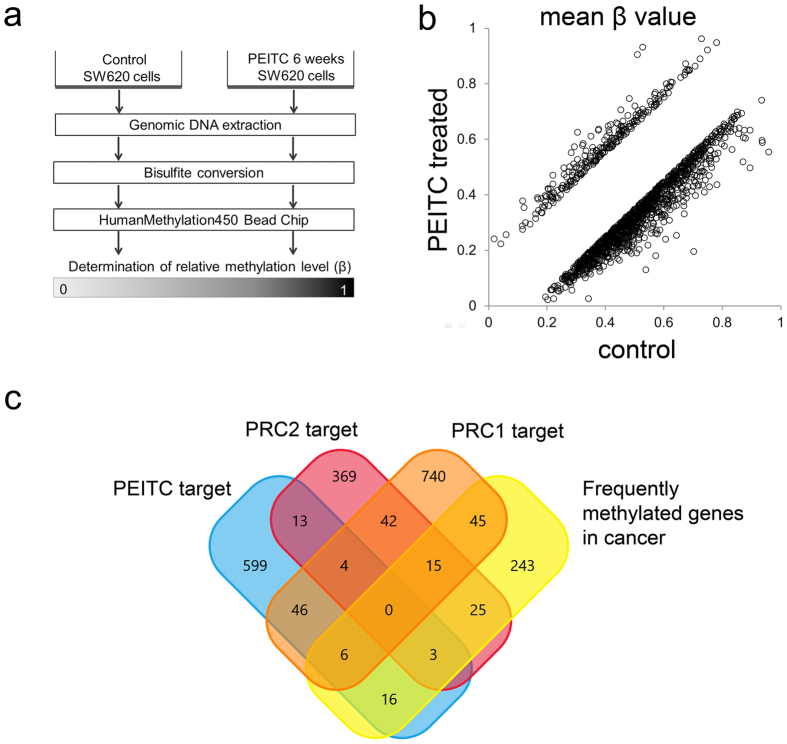
Differential DNA methylation pattern in PEITC-treated colon cancer cells. (**a**) Schematic summary of DNA methylation analysis. Genomic DNA was extracted from SW620 cells that had been treated with either 2.5 μM PEITC or DMSO only for 6 weeks and then subjected to bisulfite conversion. The Illumina Infinium HumanMethylation450 bead chip was used for determination of relative methylation levels. (**b**) Differentially methylated genes (difference > |0.15|) were identified by comparing methylation intensity (mean β value) between SW620-CON and SW620-PEITC cells. (**c**) Venn diagram representing the overlap of PEITC targets with PRC1 and PRC2 targets as well as other genes that are frequently hypermethylated in cancer. Data were generated from two independent biological replicates.

As described before, properties of bioactive phytochemicals as potent inhibitors of DNA methylation were explained in reverse hypermethylation and activation of tumor suppressor genes p21^WAF1/CIP1^ and p16^INK4a^ 19,35. Therefore, we next hypothesized that PEITC-induced changes in the chromatin distribution of epigenetic writers and erasers alters DNA methylation patterns and could potentially reverse hypermethylation of tumor suppressor genes. To assess this possibility, we extracted genomic DNA from SW620-CON and SW620-PEITC cells followed by bisulfite conversion to facilitate detection of unmethylated versus methylated cytosines and performed global analyses of methylation patterns using the Illumina Infinium platform (HumanMethylation450) which targets 466824 methylation sites across the genome^36^ (Fig. 5a). Comparison of methylation intensity (mean β-values) of the 466824 probes between SW620-CON and SW620-PEITC cells revealed differential methylation of 1451 total sites, spanning 842 annotated genes (β-value difference > |0.15|; Fig. 5b). Of these, 1203 sites/687 genes displayed more extensive DNA methylation in SW620-CON cells, whereas only 248 sites/155 genes were more highly methylated in PEITC-treated tumor cells (Supplementary Table 2).

Since the PEITC-treated cancer cells displayed reduced growth in colony formation assays *in vitro* and decreased tumorigenic potential in the mouse xenograft model *in vivo*, we next assessed whether PEITC-induced epigenetic changes could influence the methylation status of key oncogenes and/or tumor suppressor genes. When we analyzed the genes that were hypomethylated in PEITC-treated cells, we observed that these included several targets of polycomb repressive complexes (PRCs), consistent with our earlier proteomic data which revealed decreased PRC binding to heterochromatin in PEITC-treated cells. Indeed, when compared a list of 687 genes, which hypomethylated in SW620-PEITC cells, against those are either known targets of PRC1/PRC2 or frequently methylated/hypermethylated in cancer^37^,^38^,^39^, we observed that 88 of these (12.8%) exhibited decreased methylation in SW620-PEITC cells (Fig. 5c and Supplementary Table 3 and 4). Since DNA hypomethylation can lead to reactivation of silenced genes, these data support the concept that the anti-tumor effects of PEITC exposure are mediated via changes in DNA methylation pattern at known anti-cancer gene loci.

### Suppression of DNA methylation in PEITC-treated tumor cells is associated with down-regulation of Polycomb group complex

**Figure 6:**
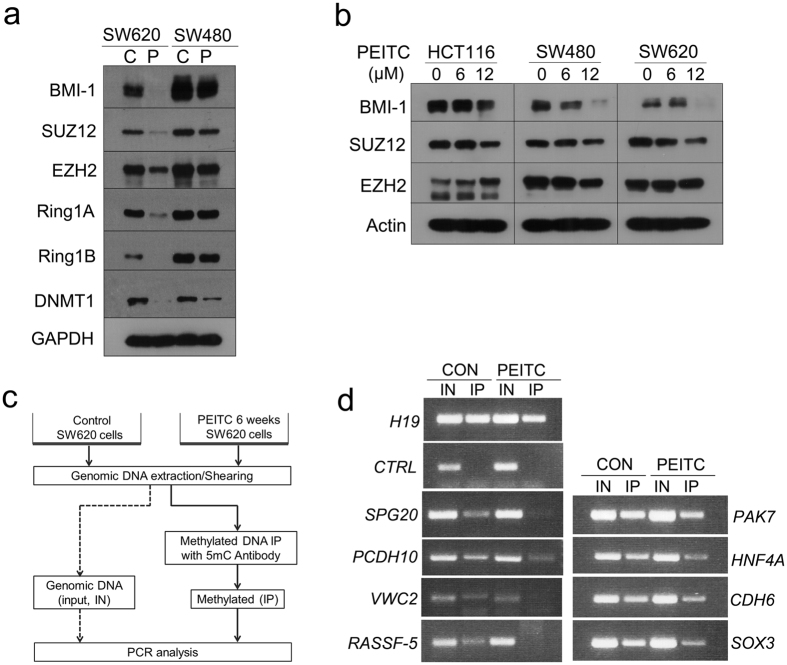
PEITC-induced hypomethylation and down-regulation of PcG complex expression. (**a**) Western blot analysis of PcG complex proteins in SW620 or SW480 cells after 6 weeks treatment with DMSO (C) or 2.5 μM PEITC (P). (**b**) Western blot analysis of PcG complex proteins in HCT116, SW620, or SW480 cells treated with various concentrations of PEITC for 48 h. (**c**) Experimental scheme of methylated DNA immunoprecipitation (MeDIP) analysis. Genomic DNA was extracted from SW620-CON or SW620-PEITC cells and then immunoprecipitated using the methylation-specific antibody 5 mC. Genes that overlapped with PcG targets and displayed differential methylation between the immunoprecipitates of SW620-CON and SW620-PEITC cells were then subjected to amplification using MeDIP-specific PCR primers that covered CpG island regions and potentially methylated sequences (**d**). Primers used are detailed in Supplementary Table 5. IN, input; IP, immunoprecipitation.

Having identified that Polycomb-group (PcG) target genes are hypermethylated in SW620 cells but display reduced methylation after long-term PEITC exposure (Fig. 5c), we next assessed whether the anti-tumor effects of PEITC are mediated via effects on PcG function. First, we analyzed whether prolonged PEITC exposure down-regulates protein levels of the PcG complex, which plays an essential role in epigenetic maintenance of the repressive chromatin mark H3K27me3^37^. As shown in Fig. 6a, western blot analyses confirmed that 6 weeks exposure to 2.5 μM PEITC was associated with substantial down-regulation of PcG complex proteins including BMI-1 (B cell-specific Moloney murine leukemia virus integration site 1), SUZ12 (suppressor of zeste 12 homolog), EZH2 (enhancer of zeste homolog 2), Ring1A, and Ring1B. Intriguingly, this PEITC-induced decrease in expression of PcG complex proteins was more pronounced in metastatic SW620 cells than in non-metastatic SW480 cells. While short-term exposure to high concentrations of PEITC (6–12 μM for 48 h) was sufficient to reduce tumor cell expression of BMI-1 (Fig. 6b), marked decreases in the expression of key PcG complex proteins SUZ12 and EZH2 were only observed after long-term treatment (2.5 μM PEITC for 6 weeks). These data indicate that prolonged exposure to BDCs is likely required to obtain the full benefit of any anti-tumor effects. We next assessed whether this PEITC influence on PcG complex expression is related with changes in gene methylation profile. To do this, we performed methyl-DNA immunoprecipitation (MeDIP) combined with PCR amplification of candidate PcG target genes that were identified in the methylation array, including *SPG20* (Spastic Paraplegia 20), *PAK7* (p21 protein activated kinase 7), *PCDH10* (Protocadherin 10), *HNF4A* (Hepatocyte Nuclear Factor 4), *VWC2* (von Willebrand factor C domain containing 2), *CDH6* (Cadherin 6), *RASSF5* (Ras association domain-containing protein 5), and *SOX3* (SRY-related HMG-box 3) (Fig. 6c). The MeDIP-PCR results identified multiple genes enriched in SW620-CON cells as being substantially decreased in SW620-PEITC cells, suggesting that these PcG targets are hypomethylated after PEITC treatment (Fig. 6d). These data support the concept that long-term PEITC treatment leads to hypomethylation of PcG target genes that are typically hypermethylated in cancer, such as *SPG20, PCDH10, HNF4A, VWC2, CDH6*, and *RASSF5*.

### Tumor cell overexpression of BMI-1 attenuates the cytotoxic effects of PEITC

**Figure 7:**
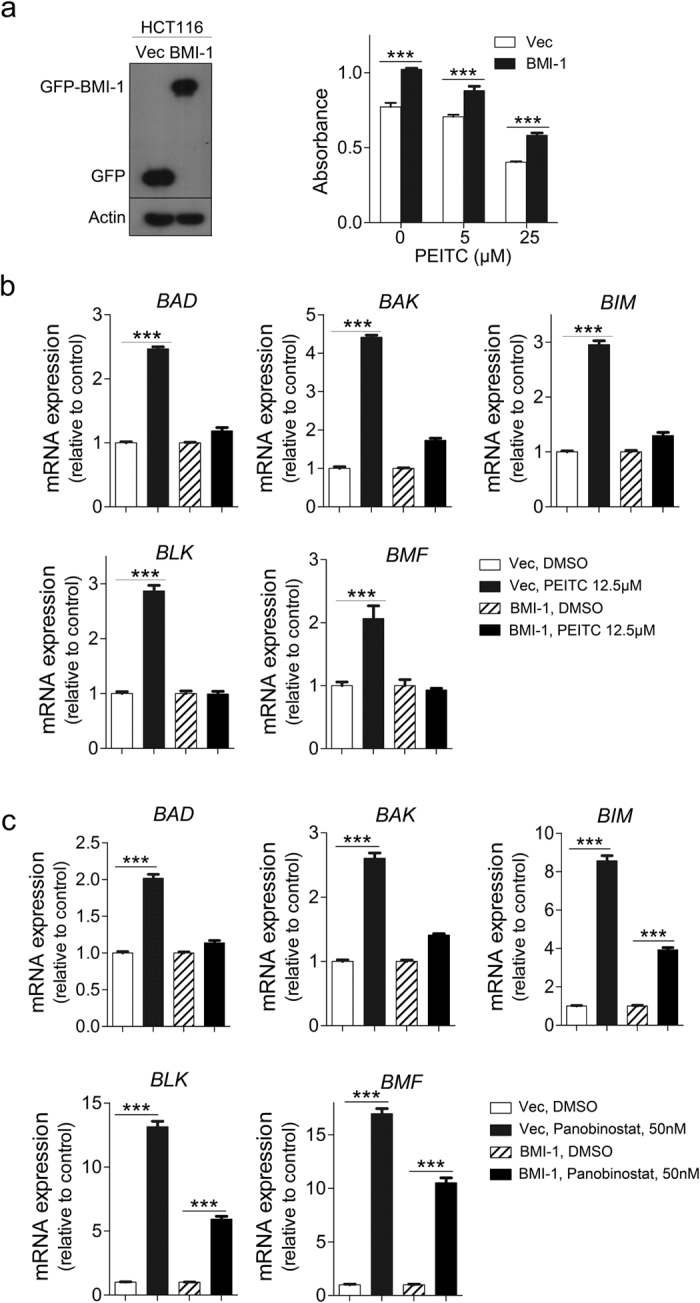
BMI-1 overexpression protects HCT116 tumor cells against PEITC-induced cytotoxicity and apoptosis. (**a**) Left panel: Western blot analysis of HCT116 cells expressing GFP only (Vec) or overexpressing BMI-1 (BMI-1). Actin was used as the loading control. Right panel: MTT assay demonstrating that HCT116-BMI-1 cells are partially protected against the cytotoxic effects of PEITC treatment. Error bars represent the mean ± s.d., n = 4. Statistical significance between HCT116-Vec and HCT116-BMI-1 cells was estimated using Student t-test (PEITC 0 μM, *p* = *0.0001*; 5 μM, *p* = *0.0017*; 25 μM, p < *0.0001*). (**b**) Significant upregulation of pro-apoptotic gene expression in HCT116-Vec but not HCT116-BMI-1 cells after 24 h treatment with 12.5 μM PEITC (or DMSO-only control). Error bars represent the mean ± s.d., n = 3. (**c**) Pro-apoptotic gene expression in HCT116-BMI-1 cells (or HCT116-Vec control) after 24 h treatment with 50 nM panobinostat or DMSO-only vehicle control. Expression levels of pro-apoptotic genes were upregulated by drug treatment in HCT116-Vec cells but not in HCT116-BMI-1 cells, which displayed mRNA expression levels of *BAD/BAK* comparable to the DMSO-only control and exhibited only limited induction of *BIM, BLK,* and *BMF* genes. Error bars represent the mean ± s.d., n = 3. Statistical differences between groups were determined using Student’s t-test (***p < *0.001*).

Since we determined that tumor restriction by PEITC is associated with down-regulation of PcG complex proteins and hypomethylation of PcG target genes, we next assessed whether these anti-cancer effects could be reversed by restoring PcG function in these cells. To do this, we overexpressed the PcG component BMI-1 in HCT116 cells and assessed tumor cell survival in MTT assays. In these experiments, HCT116 cells that overexpressed BMI-1 (HCT116-BMI-1) exhibited ~30% more growth over a 48 h period than did control HCT116 cells expressing GFP only (HCT116-Vec) (*p* = *0.0001*, Fig. 7a). Intriguingly, HCT116-BMI-1 cells maintained higher viability even after exposed to high concentrations of PEITC for 48 h in culture, suggesting that tumor cell overexpression of BMI-1 can effectively suppress the cytotoxic effects of PEITC (25 μM, *p* < *0.0001*). Since PEITC treatment up-regulated the expression of pro-apoptotic genes in SW620 cells and BMI-1 overexpression was able to rescue the viability of tumor cells undergoing PEITC treatment (Fig. 7a,b), we next investigated whether enhanced expression of BMI-1 could protect HCT116 cells against PEITC-mediated apoptosis. We therefore used qRT-PCR to measure the expression of pro-apoptotic genes (*BAD, BAX, BIM, BLK,* and *BMF*) in HCT116-Vec cells or HCT116-BMI-1 cells treated with 12.5 μM PEITC for 24 h (Fig. 7b). Indeed, we observed that PEITC treatment was unable to increase expression of pro-apoptotic genes in HCT116-BMI-1 cells, indicating that BMI-1 overexpression increases cancer cell survival by blocking the effects of PEITC. Having previously established that PEITC treatment can also block HDAC binding to euchromatin, we next assessed whether BMI-1 overexpression was able to protect tumor cells against treatment with the pan-HDAC inhibitor panobinostat^13^. While panobinostat-treated HCT116-Vec cells displayed significant upregulation of pro-apoptotic genes including *BAD, BAX, BIM, BLK,* and *BMF,* HCT116-BMI-1 cells were relatively protected against the effects of panobinostat treatment, as evidenced by mRNA expression levels of *BAD* and *BAK* that were comparable with the DMSO-only vehicle control. While panobinostat did induce modest upregulation of *BIM, BLK*, and *BMF* in HCT116-BMI-1 cells, expression levels of these genes were still relatively lower than those detected in HCT16-Vec cells subjected to the same treatment (Fig. 7c). Together, these data suggest that the anti-tumor effects of PEITC can be overcome by pro-survival signals mediated by overexpression of BMI-1.

## Discussion

Cancer risk is known to be modified by dietary intake of bioactive phytochemicals, which can be accumulated and affect epigenetic alterations through modulation of DNA/histone modification. Epigenetic regulation by phytochemicals including the cruciferous vegetable derivatives sulforaphane (SFN), phenethyl isothiocyanate (PEITC) and indol-3-carbinol were studied in several cancer models^6^,^40^,^41^,^42^, but the epigenetic basis of these anti-tumor effects has still remained elusive. The current study presents evidence that PEITC regulates the chromatin binding profile of key epigenetic writers/erasers and PcG complexes to restrict the development of tumors.

Defects in epigenetic regulation of gene expression are increasingly being recognized as critical mediators of cancer development^43^, hence therapeutic targeting of HDAC function is being extensively evaluated in clinical trials^13^,^44^. While several HDAC inhibitors have demonstrated clear anti-tumor benefits such as cell cycle arrest, inhibition of proliferation, and apoptosis induction^13^, these same effects could potentially also be achieved via dietary interventions without the risk of potentially severe off-target side effects^6^,^41^. Indeed, previous longitudinal studies of associations between diet and cancer incidence have already confirmed potent anti-tumor effects of consuming bioactive dietary components (BDCs) such as indoles, nitriles, and isothiocyanates^4^,^41^. Several of these compounds can elicit host immune protection against tumors via signaling through the aryl hydrocarbon receptor pathway^45^,^46^, but direct effects on the epigenetic profile of transformed host cells have also been proposed.

In order to better understand the epigenetic basis of BDC-mediated protection against tumors, we established a cell line model of habitual consumption of cruciferous vegetables by subjecting SW620 colon cancer cells to physiologically relevant concentration of PEITC for 6 weeks. Blood plasma concentrations of PEITC after consumption of glucosinolate-rich watercress or broccoli sprout isothiocyanates in human have been reported to be reached ~2.3 μM, which was comparable to those used in our *in vitro* study^47^,^48^. PEITC-treated cancer cells displayed reduced growth and decreased tumorigenic potential even after subsequent PEITC withdrawal, thus indicating that sustained exposure to PEITC induces stable and heritable changes in tumor cell phenotype. Intriguingly, pro-apoptotic genes were up-regulated 20–50-fold in tumor cells after prolonged treatment with low-dose PEITC (2.5 μM), whereas the same genes displayed only 2–3-fold induction after a brief exposure to high-dose PEITC (12.5 μM). When we assessed the molecular basis of these effects using LC-MS-driven chromatome profiling, we observed that several key epigenetic regulators were dynamically modulated by PEITC exposure. Long-term treatment with PEITC was associated with preferential binding of HATs to active euchromatin, whereas HDACs were redistributed to regions of heterochromatin.We therefore proceeded to analyze how PEITC exposure altered DNA methylation patterns across the genome and identified genes that were differentially methylated after treatment. We observed that long-term exposure to PEITC was associated with hypomethylation of known targets of the PcG complex and other genes that are typically hypermethylated in cancer. Consistent with these findings, we also detected substantial down-regulation of PcG complex protein expression in PEITC-treated cancer cells. Conversely, overexpression of PcG complex member BMI-1 impaired the up-regulation of pro-apoptotic genes in HCT116 cells following treatment with PEITC or the pan-HDAC inhibitor panobinostat. Given that PEITC treatment also suppressed HDAC binding to euchromatin, suggesting that PEITC restricts tumor growth by preventing HDAC suppression of pro-apoptotic genes.

Together, our data demonstrate that prolonged exposure to an archetypal BDC can induce stable changes in tumor cell expression of epigenetic regulators and pro-apoptotic genes. In particular, our results indicate that the chemopreventive effects of PEITC exposure are associated with stable changes in HDAC distribution and hypomethylation of known targets of the PcG complex and other genes that are frequently methylated in cancer.

Much attention on chemopreventive effects of phytochemicals also bring the issue of interindividual differences in absorption, metabolism, and disposition of phytochemicals. It has been reported that genetic polymorphism of those key metabolizing enzymes including glutathione S-transferases, UDP-glucuronosyltransferases, and cytochrome P450 2E1 may partly account for variation in cancer risk^49^,^50^. Therefore, more comprehensive evaluation of the role of these dietary compounds in cancer prevention is required. By elucidating the mechanistic interactions between BDCs, chromatome regulators, epigenetic marks, and anti-tumor effects, these data might be useful to assist the exploitation of BDC biology for understanding of cancer chemoprevention.

## Materials and Methods

### Cell lines, plasmid, and treatments

Colorectal cancer cell lines SW620, SW480, and HCT116 were obtained from American Type Culture Collection. Cells were maintained in RPMI medium supplemented with 10% FBS and 1% penicillin/streptomycin and then cultured in the presence of 2.5 μM PEITC (or 0.01% DMSO vehicle-only) for a total duration of 6 weeks. In some experiments, the cells were subjected to various doses of PEITC (0–20 μM) for the indicated times. The pEGFP-BMI-1 plasmid was generated by insertion of the full-length human *BMI-1* cDNA into the *Xho*I/*BamH*I sites of a pEGFP-C1 vector.

### Cell viability analysis

Cell viability was determined by MTT assay using 5 × 10^3^ cells/well seeded into 96-well plates and then incubated with various concentrations of PEITC for the indicated times. For colony formation assays, SW620 cells were treated with 2.5 μM PEITC (or DMSO only) for 6 weeks before being transferred into a 6-well plate and cultured for a further 21 days in the presence or absence of 2.5 μM of PEITC. Colony formation was then quantified by crystal violet staining and dye solubilization in DMSO prior to absorbance measurement using a microplate reader.

### PEITC-mediated anti-cancer effects in mouse xenograft model

All animal studies were approved by an Institutional Animal Care and Use Committee (IACUC, ARF-SBS/NIE-A0242) and were performed in accordance with approved guidelines and regulations of the Animal Facility Center of School of Biological Scienecs, Nanyang Technological University, Singapore. NCr nude mice were obtained from InVivos, Pte. Ltd. (Singapore). We performed subcutaneous (s.c.) injection of 1 × 10^6^ SW620-CON (n = 6) or SW620-PEITC (n = 6) cancer cells into either the left or right flank of NCr nude mice and monitored tumor size on both sides of animals with caliper. The tumor size reached 1.5 cm in diameter, the mice were euthanized as per guideline.

### Preparation of euchromatin/heterochromatin-associated proteins

SW620-CON and SW620-PEITC cells were subjected to partial MNase treatment to enable differential extraction of euchromatin- and heterochromatin-associated proteins as described previously^30^. A total of 100 μg protein from each chromatin fraction was separated on an 8–20% gradient SDS-PAGE gel and subjected to in-gel digestion before labelling the resultant peptides using the TMT-6plex Isobaric Label Reagent Set (Thermo Scientific, Rockford, IL, USA) according to the manufacturer’s protocol. Details of the labeling scheme used are provided in the supplementary data. The labeled samples were combined prior to fractionation on a Xbridge™ C18 column (4.6 × 250 mm, Waters, Milford, MA, USA) and subsequent analysis by LC-MS/MS.

### Liquid chromatography with tandem mass spectroscopy

The fractionated peptides were separated and analyzed using a Dionex Ultimate 3000 RSLCnano system coupled to a Q Exactive instrument (Thermo Fisher Scientific, MA, USA). Separation was performed on a Dionex EASY-Spray 75 μm × 10 cm column packed with PepMap C18 3 μm, 100 Å (Thermo Fisher Scientific) using solvent A (0.1% formic acid in 5% ACN) and solvent B (0.1% formic acid in 90% ACN) at flow rate of 300 nL/min with a 60 min gradient. Peptides were then analyzed on a Q Exactive apparatus with an EASY nanospray source (Thermo Fisher Scientific) at an electrospray potential of 1.5 kV. A full MS scan (350–1,600 m/z range) was acquired at a resolution of 70,000 at m/z 200 and a maximum ion accumulation time of 100 ms. Dynamic exclusion was set as 15 s. The resolution of the higher energy collisional dissociation (HCD) spectra was set to 17,500 at m/z 200. The automatic gain control (AGC) settings of the full MS scan and the MS2 scan were 3E6 and 2E5, respectively. The 10 most intense ions above the 2,000 count threshold were selected for fragmentation in HCD, with a maximum ion accumulation time of 100 ms. An isolation width of 2 was used for MS2. Single and unassigned charged ions were excluded from MS/MS. For HCD, the normalized collision energy was set to 28%. The underfill ratio was defined as 0.2%. Raw data files from the three technical replicates were processed and searched using Proteome Discoverer 1.2 (Thermo Fisher Scientific). The raw LC-MS/MS data files were loaded into Spectrum Files (default parameters set in Spectrum Selector) and TMT 6-plex was selected for the Reporter Ion Quantifier. The SEQUEST algorithm was then used for data searching to identify proteins using the following parameters; missed cleavage of two; dynamic modifications were oxidation (+15.995 Da) (M), deamidation (+0.984 Da) (N) and phosphorylation (+79.9966 Da) (S, T, Y). The static modifications were TMT-6plex (+229.163 Da) (any N-terminus and K) and Carbamidomethyl (+57 Da) (C). The false discovery rate for protein identification was <1%. The ‘Normalize on Protein Median’ function was used for protein quantitation, and fold-change log2 value > |0.5| was defined as differential expression.

### Western blot analysis

Cells were washed in ice-cold PBS and lysed in modified RIPA buffer (50 mM Tris–HCl, 150 mM NaCl, 1% NP‐40, pH 8.0, 1× protease inhibitor cocktail, and phosphatase inhibitors). Lysates were clarified by centrifugation (16,000 × g, 30 min) and subjected to western blotting using the indicated primary antibodies at 1:1000 dilution. Protein-antibody conjugates were visualized using a chemiluminescent detection kit (ThermoScientific). Antibodies against BMI-1, SUZ12, EZH2, Ring1A, Ring1B, and DNMT1 were purchased from Cell Signaling Technologies (Danvers, MA, USA).

### RNA extraction and quantitative real-time PCR

RNA extraction was performed using Nucleospin RNA kits (MACHEREY-NAGEL GmbH & Co.) according to the manufacturer’s protocol. Quantitative real-time PCR was performed by CFX96 Real-Time PCR Detection System (Bio-Rad) with KAPA SYBR® FAST qPCR Master Mix. Actin or 18sRNA were used as internal controls. The primer sequences used for real-time PCR are provided in the Supplementary Information (Table S6).

### Soft-agar colony formation assay

Colon cancer cells (1 × 10^3^ cells/well) were suspended in culture medium containing 0.3% agar (Sigma Chemical) and plated over a lower layer of 0.7% agar-medium mixture. Viable colonies > 0.5 mm diameter were counted after 14 days culture.

### Epigenome-wide DNA methylation assays

Genomic DNA was treated with sodium bisulfite using the Zymo EZ-DNA kit (Zymo Research, Orange, CA, USA) according to manufacturer’s instructions and then hybridized to Illumina Infinium HumanMethylation450 (HM450) BeadChips (Illumina, San Diego, CA, USA). The chips were scanned with Illumina iScan and the data extracted using the methylumi package in R on Bioconductor. Methylation intensity was reported as β-values [β = intensity of the methylated allele/(intensity of the unmethylated allele + intensity of the methylated allele)]. Probes were regarded as ‘failed’ when there was no significant difference from background noise after background correction (P > 0.01).

### Methylated DNA Immunoprecipitation (MeDIP) and PCR analysis

Differentially methylated regions that overlapped with known PcG target genes were validated using MeDIP followed by PCR analysis. Enrichment of methylated DNA was carried out using anti-5-methylcytosine (5-mC) antibody (Zymo Research, Orange, CA, USA). Briefly, genomic DNA was sheared by sonication to obtain fragments between 200–800 bp and the methylated DNA captured using a modified antibody immunoprecipitation protocol. Final eluates were obtained by re-suspending the protein A/G-coupled Sepharose beads in a 200 μL of TE buffer containing 2 μL proteinase K (20 mg/ml) and then subsequently incubated at 55 °C with 800 rpm agitation for 90 min. Methyl enrichment in the samples was confirmed by PCR analysis. Fully methylated DNA and completely unmethylated DNA samples (human HCT116 DKO methylated & non-methylated DNA set, Zymo Research) were used as experimental controls. After amplification, products were resolved on 2% agarose gels containing ethidium bromide and then visualized under UV transillumination.

### Secreted alkaline phosphatase (SEAP) assay

AP-1, CRE, and NFkB SEAP reporter vectors were purchased from Clontech (Palo Alto, CA, USA). SW620-CON and SW620-PEITC cells were transfected with 200 ng of the indicated SEAP vector and incubated for 24 h. The culture supernatant was collected, heated at 65 °C for 30 min, and then assayed for alkaline phosphatase activity as follows; 30 μl of supernatant was incubated with 120 μl of assay buffer for 5 min, at which time CSPD substrate (1:20 dilution) was added and the samples were read on a TECAN microplate reader (Maennedorf, Switzerland)^34^.

### Cell cycle analysis

The DNA content of cultured cells was quantitated by staining with propidium iodide (PI) and analysed by flow cytometry (BDLSR11, Becton Dickinson, San Jose, CA). Briefly, cells were harvested with PBS and fixed with cold 70% ethanol at 4 °C for 30 min. The cells were washed with PBS and then resuspended in 500 μl of PI staining solution and incubated for 30 min at room temperature. Samples were then examined and analysed for cell cycle phase (Modfit LT2.0, Becton Dickinson).

### Statistical analysis

Statistical analysis was done using SPSS18 statistical software (version 18.0; SPSS, Chicago, IL, USA). Statistical differences between variables were determined by student’s t-test and p < 0.05 was considered.

## Additional Information

**How to cite this article**: Park, J. E. *et al*. Dietary phytochemical PEITC restricts tumor development via modulation of epigenetic writers and erasers. *Sci. Rep.* **7**, 40569; doi: 10.1038/srep40569 (2017).

**Publisher's note:** Springer Nature remains neutral with regard to jurisdictional claims in published maps and institutional affiliations.

## Supplementary Material

Supplementary Information

Supplementary Table S1

Supplementary Table S2

Supplementary Table S3

Supplementary Table S4
