# Supplementary material for: Dietary phytochemical PEITC restricts tumor development via modulation of epigenetic writers and erasers
Source: Sci Rep. 2017 Jan 12;7:40569. doi: 10.1038/srep40569 (PMC5228035; doi:10.1038/srep40569)
Supplement: Supplementary Information [file srep40569-s1.pdf]

Dietary phytochemical PEITC restricts tumor development via modulation of epigenetic writers and erasers

Jung Eun Park[1], Sun Yang[1], Sai Kiang Lim[2], James P Tam[1], Matthijs Dekker[3], Hong Chen[4], and Siu Kwan Sze[1,]*

Supplementary Information

Supplementary Figure 1. Effects of PEITC on cell viability of colon cancer cells.

Supplementary Figure 2. Reduced growth of PEITC-treated tumors in a mouse xenograft model.

Supplementary Table 1. List of chromatin-associated proteins identified by ProteomeDiscoverer v1.4 search from 6-plex TMT experiment of 2.5 µM PEITC- or DMSO-treated SW620 cells (excel file).

Supplementary Table 2. List of differentially regulated chromatome after PEITC treatment for 6 weeks (excel file).

Supplementary Table 3. PEITC-induced differentially methylated genes in SW620 cells (excel file).

Supplementary Table 4. Lists of PEITC-, PRC1-, or PRC2 target genes and frequently methylated genes in cancer (excel file).

Supplementary Table 5. Common PEITC target genes which are overlapped with PRC1, PRC2, or frequently methylated cancer specific genes.

Supplementary Table 6. MeDIP primers.

Supplementary Table 7. Pro-apoptotic gene primers.

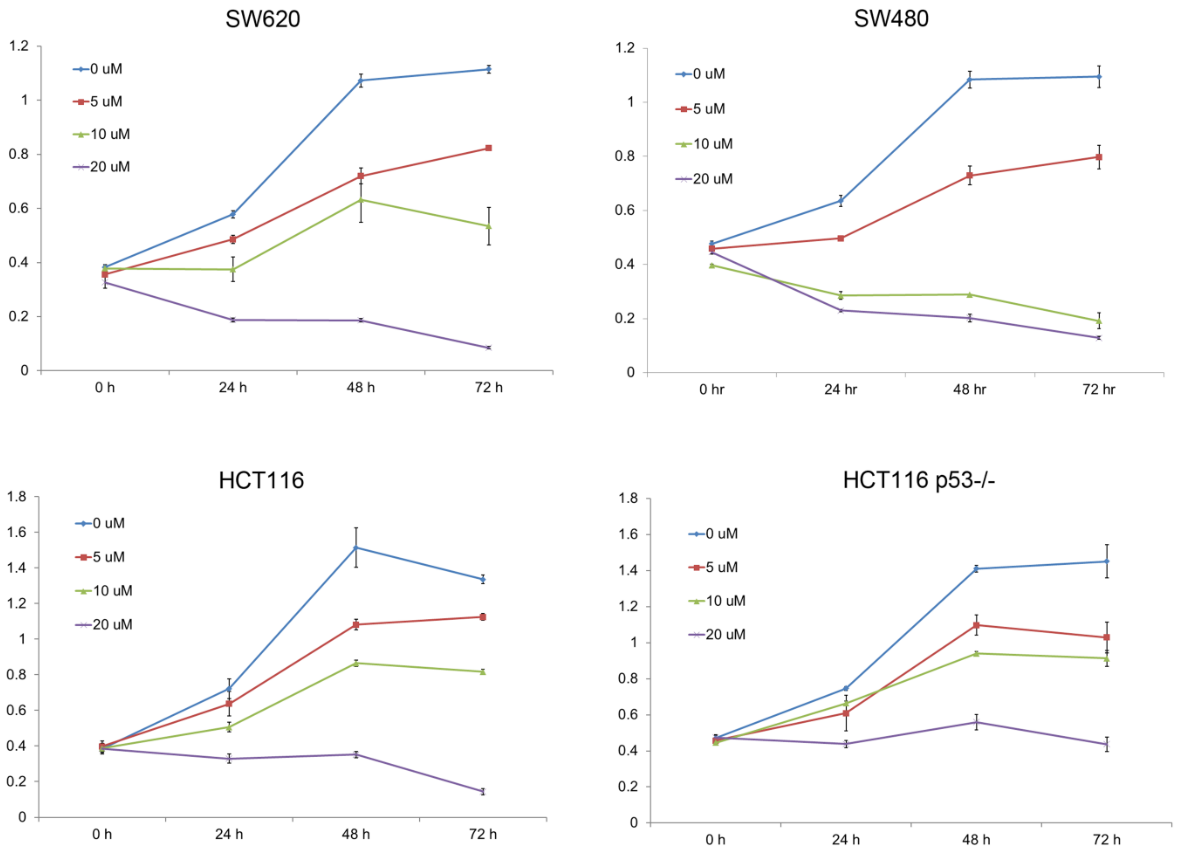

Supplementary Figure 1. Effects of PEITC on cell viability of colon cancer cells.

MTT assay showed cell viability of colon cancer cells after treatment with PEITC for the various time points. Error bars represent the mean ± s.d. from three independent experiments.

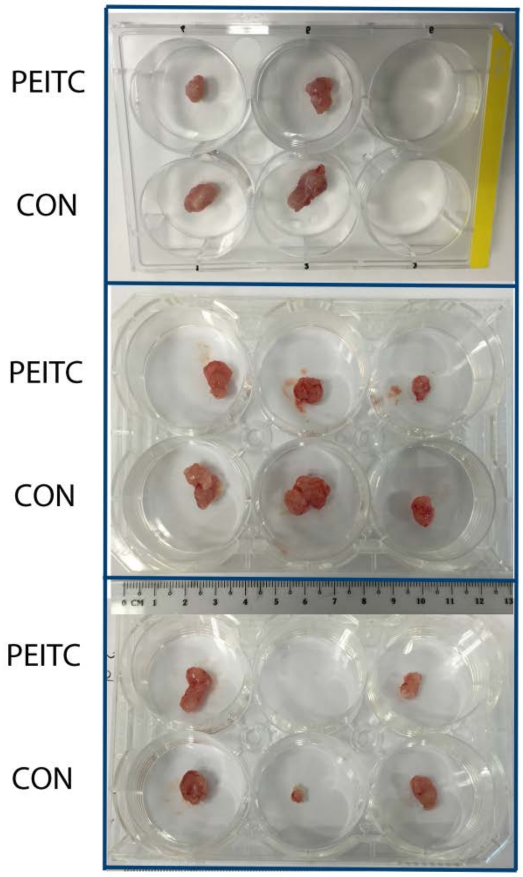

Supplementary Figure 2. Reduction of tumor formation in mouse xenograft model. Mouse xenograft model showed a reduction of tumor formation in SW620-PEITC cells compared to control cells. SW620 cells after treatment with DMSO (CON) or 2.5 µM PEITC (PEITC) for 6 weeks were s.c. injected into left or right flank region of NCr nude mice.

Supplementary Table 5. Common PEITC target genes which are overlapped with PRC1, PRC2, or frequently methylated cancer specific genes

Gene name	PEITC target	PRC2 target	PRC1 target	Frequently methylated gene
CDH8	o	o		
DLC1	o	o		
FEZ1	o	o		
ITGA2	o	o		
MGLL	o	o		
PAX7	o	o		
RASSF5	o	o		
TFPI2	o	o		
TRIM15	o	o		
TRPM3	o	o		
oSNL1	o	o		
WNT5A	o	o		
MORF4	o	o		
HECW2	o	o	o	
PAPPA	o	o	o	
PRKCH	o	o	o	
PTHLH	o	o	o	
CFTR	o	o		o
FBN1	o	o		o
SFRP1	o	o		o
ALCAM	o		o	
ALOX15	o		o	
B4GALNT2	o		o	
BAMBI	o		o	
CACNA1A	o		o	
CDH6	o		o	
CR2	o		o	
DLGAP2	o		o	
DPP10	o		o	
DSP	o		o	
EBF2	o		o	
ELAoL4	o		o	
EPHA3	o		o	
ESRP1	o		o	
EoX2	o		o	
FAM46C	o		o	
FLI1	o		o	
GAD1	o		o	
GALNT3	o		o	
GRM8	o		o	
HEPACAM2	o		o	
HPSE2	o		o	

HSPB3	o	o	
KCNA3	o	o	
KCTD16	o	o	
KLHL29	o	o	
LHX5	o	o	
LRRCC1	o	o	
MAML3	o	o	
MECOM	o	o	
MSX1	o	o	
PAK7	o	o	
PAX3	o	o	
PCDH10	o	o	
PCDH17	o	o	
PCDH18	o	o	
PDE1A	o	o	
PEG3	o	o	
PTH2R	o	o	
RALYL	o	o	
RIMKLB	o	o	
RUNX1T1	o	o	
RUNX2	o	o	
SCGB3A2	o	o	
WNT8B	o	o	
ZIC4	o	o	
ADCYAP1	o	o	o
EBF3	o	o	o
GDNF	o	o	o
GFI1	o	o	o
HOXA11	o	o	o
TMEFF2	o	o	o
AFF3			o
ASCL2			o
COL1A2			o
CSMD1			o
DDIT3			o
FGFR2			o
FLRT2			o
GABRB3			o
HLA-DPB1			o
HS3ST2			o
MAGEA1			o
MGMT			o
MYLK			o
PPP2R2B			o
SFRP2			o
SNRPN			o

Supplementary Table 6. MeDIP primers

Gene	Forward (5'-3)	Reverse (5'-3)
VWC2	TACATCAGAAACACGCCGGCCG	CCTGCGGAGAGAACAGCAGCAC
PCDH10	TTCTTTGCGTCTGTCCCAGGCA	ATTCACACTGTGTGGGCGCTCG
SPG20	ACTCCTTAGAGCTAGCACAAGTTGA	TGTTTGAAACAGGAAGCCTTTCTGT
PAK7	GTACGCAGCTGCTCCGGGTTTT	TCCTCCCCACCTCTGCTAGCCT
CDH6	ACCTTCGCATCCCAAGAGCTGC	AGAGCATCCCTCCCAACTCCGG
SOX3	GGGCTCTAGCTGGGCCCCTTAT	CGTGGACTCCCGCCTCCTAAGT
HNF4A	GAAGGCAGAGAGGGCACTGGGA	CTGGCACACCTGGGCACATCTG
RASSF-5	TTA ATT ATT TCG ATT GAT TC	CGA TAA CTC AAA TTC CAC G
MeDIP_H19	CGA GTG TGC GTG AGT GTG AG	GGC GTA ATG GAA TGC TTG AA
MeDIP_CTRL	GAG AGC ATT AGG GCA GAC AAA	GTT CCT CAG ACA GCC ACA TTT

Supplementary Table 7. Pro-apoptotic gene primers

Gene	Forward (5'-3')	Reverse (5'-3')
BAK	ACA CTT GCT CCC AAC CCA TTC A	AAC CAA GTC CTA AGC CCT GT
BLK	ATC TTG ATG GAG ACC CTC CTG T	TCA CTG CCC TCC ATG CAT T
BMF	ACC AGC CAG GAA GAC AAA GCT	CGA TAG CCA GCA TTG CCA TAA
BAD	AGT GAC GAG TTT GTG GAC TCC T	AGC TTT GCC GCA TCT GCG TT
BIM	GTT GCT TTG CCA TTT GGT CT	CCA CCA CTT GAT TCT TGC AG
